# Mitochondrial genome sequence variation as a useful marker for assessing genetic heterogeneity among *Cyclospora cayetanensis* isolates and source-tracking

**DOI:** 10.1186/s13071-019-3294-1

**Published:** 2019-01-21

**Authors:** Yaqiong Guo, Yuanfei Wang, Xiaolan Wang, Longxian Zhang, Ynes Ortega, Yaoyu Feng

**Affiliations:** 1 Key Laboratory of Zoonosis of Ministry of Agriculture, College of Veterinary Medicine, South China Agricultural University, Guangzhou, 510642 Guangdong China; 2 College of Animal Science and Veterinary Medicine, Henan Agricultural University, Zhengzhou, 450002 Henan China; 3 College of Agricultural and Environmental Sciences, University of Georgia, Griffin, Georgia 30223 USA

**Keywords:** *Cyclospora cayetanensis*, Mitochondrion, qPCR, Genotyping, Source-tracking

## Abstract

**Background:**

*Cyclospora cayetanensis* is an important enteric pathogen, causing diarrhea and food-borne cyclosporiasis outbreaks. For effective outbreak identification and investigation, it is essential to rapidly assess the genetic heterogeneity of *C. cayetanensis* specimens from cluster cases and identify the likely occurrence of outbreaks.

**Methods:**

In this study, we developed a quantitative PCR (qPCR) targeting the polymorphic link region between copies of the mitochondrial genome of *C. cayetanensis*, and evaluated the genetic heterogeneity among 36 specimens from six countries using melt curve, gel electrophoresis, and sequence analyses of the qPCR products.

**Results:**

All specimens were amplified successfully in the qPCR and produced melt peaks with different Tm values in the melt curve analysis. In gel electrophoresis of the qPCR products, the specimens yielded bands of variable sizes. Nine genotypes were identified by DNA sequencing of the qPCR products. Geographical segregation of genotypes was observed among specimens analyzed, which could be useful in geographical source-tracking.

**Conclusions:**

The length and nucleotide sequence variations in the mitochondrial genome marker allow rapid assessment of the genetic heterogeneity among *C. cayetanensis* specimens by melt curve, gel electrophoresis, or DNA sequence analysis of qPCR products. The sequence data generated could be helpful in the initial source-tracking of the pathogen.

## Background

*Cyclospora* spp. are protozoan parasites that mainly cause diarrhea in humans and animals. Of the more than 20 known *Cyclospora* species, *Cyclospora cayetanensis* is the only one infecting humans. Cyclosporiasis caused by this species presents a serious challenge to food safety [1, 2]. In some resource-poor countries, cyclosporiasis is endemic and causes prolonged diarrhea in children and immunodeficient patients [3, 4]. With increased globalization in tourism and food supply, the chance of *C. cayetanensis* spreading from disease-endemic regions to other areas has increased substantially. Large outbreaks of cyclosporiasis have been reported almost annually in North America and some European countries during the last 20 years [2, 5, 6], mostly associated with contaminated food imported from cyclosporiasis-endemic counties [7]. The food-borne and imported nature of cyclosporiasis in industrialized nations suggests the need of development of molecular diagnostic tools for the identification and investigation of outbreaks.

The recent whole genome sequencing of *C. cayetanensis* has facilitated the development of genotyping tools for this pathogen [8–10]. To improve the investigation of cyclosporiasis outbreaks, a high-resolution multilocus sequence typing (MLST) tool has been developed recently for *C. cayetanensis* based on whole genome sequence data [11, 12]. Like most other eukaryotic parasites, *C. cayetanensis* has a mitochondrial genome [10, 13, 14]. As mitochondrial genomes are evolving more quickly than nuclear genomes [15], molecular typing assays developed based on mitochondrial sequences generally have high resolution. Currently, mitochondrial sequences have been used in genotyping and geographical source-tracking of *Plasmodium falciparum* and *P. vivax* [16, 17].

In a previous study, eight single-nucleotide variants (SNVs) and one 7-bp multiple-nucleotide variant (MNV) in the mitochondrial genomes were detected between two *C. cayetanensis* isolates from China and the USA, making the mitochondrial genome a potential marker for the development of a genotyping tool [13]. As the mitochondrial genome of *C. cayetanensis* has about 500 copies per cell [13], molecular diagnostic tools targeting mitochondrial sequences may have the additional advantage of higher detection sensitivity than those targeting nuclear genes.

In this study, we developed a quantitative PCR (qPCR) targeting the polymorphic link region of concatenated copies of mitochondrial genomes of *C. cayetanensis*, and assessed genetic heterogeneity among specimens from several countries by melt curve and electrophoresis analyses, and the ability for geographical source-tracking by DNA sequence analysis of the qPCR products.

## Methods

### Specimens

Thirty-six *C. cayetanensis*-positive fecal specimens were used in this study, including those from China (*n* = 21), Peru (*n* = 8), Nepal (*n* = 3), Indonesia (*n* = 2), Guatemala (*n* = 1) and Spain (*n* = 1) (Table 1). Specimens from China were collected from Henan, China, in a published epidemiological study of cryptosporidiosis [18]; specimens from Peru were collected from a small community (Pampas de San Juan de Miraflores) in Lima, Peru [19]; and specimens from Nepal, Indonesia, Guatemala and Spain were collected from sporadic cases in these countries. DNA was extracted from each specimen by using the FastDNA Spin Kit for Soil (MP Biomedicals, Carlsbad, CA, USA) and stored at -20 °C until molecular analyses.

**Table 1:** Specimens used in this study and their genotypes obtained by melt curve analysis of mitochondrial qPCR products

Genotype	PCR product size (bp)	No. of 15-bp repeats	No. of specimens	Source of specimens	Ct value (mean ± SD)	Tm value (mean ± SD)
CM-1	312	1	1	China, Kaifeng	31.23	78.32
CM-2	312	1	19	China, Zhengzhou	26.99 ± 2.87	78.10 ± 0.24
CM-3	357	4	6	Peru	26.38 ± 1.73	75.86 ± 0.22
CM-4	372	5	2	Peru	26.40 ± 1.73	75.43 ± 0.07
CM-5	342	3	1	Nepal	24.64	76.89
CM-6	342	3	2	Nepal	27.15 ± 282	76.22 ± 0.06
CM-7	327	2	2	Indonesia	25.64 ± 4.57	77.19 ± 0.78
CM-8	357	4	1	Guatemala	31.23	75.81
CM-9	357	4	1	Spain	22.35	75.95
Uninterpretable sequence	na	na	1	China, Zhengzhou	29.28	77.34

*Abbreviation*: *na* not available

### Development of a mitochondrial DNA-based qPCR

Based on results of the previous comparison of the mitochondrial genomes of *C. cayetanensis* isolates from China (CHN_HEN01; KP796149) and the USA (Cyclo_CDC_2013; KP658101) [13], we designed a set of qPCR primers that amplifies a ~357 bp link region between different copies of the mitochondrial genome. The forward and reverse primers were designed based on conservative sequences of SSU rRNA fragments, SSU/11 and SSU/4, respectively: Cyc-Mito-F1 (5'-GAG CGG TGT GTT TAA GGC AA-3') and Cyc-Mito-R1 (5'-CTG CTG GGA CTT TGT CTC TTG T-3'). The amplicon included the 7-bp MNV and one of the eight SNVs previously identified in the entire mitochondrial genome [13].

The total volume of the qPCR preparation was 50 μl, which contained 1 μl of DNA, 200 μM deoxynucleotide triphosphate, 3 mM MgCl_2_, 500 nM forward and reverse primers, 1× GeneAmp PCR buffer (Applied Biosystems, Foster City, CA, USA), 1× EvaGreen (Biotium, Hayward, CA, USA), 2.5 U of *Taq* polymerase (Promega, Madison, WI, USA) and 400 ng/μl of nonacetylated bovine serum albumin (Sigma-Aldrich, St. Louis, MO, USA). Amplification was performed on a Light Cycler® 480 system (Roche, Mannheim, Germany) with an initial denaturation at 95 °C for 3 min, followed by 50 cycles of amplification consisting of denaturation at 95 °C for 5 s, annealing at 58 °C for 15 s, and extension at 72°C for 15 s. This was followed with a melt curve analysis consisting of 95 °C for 10 s, 48 °C for 30 s, and 0.1 °C melt increments from 48 to 80 °C, and a final cooling at 40°C for 30 s. Data collection was done at each increment of the melt curve analysis. Amplification and melt curves were generated by using the Light Cycler® 480 software (v.1.5.1.62 SP3), with the threshold values and melt temperatures calculated for each specimen.

### Gel electrophoresis and sequence analyses

To confirm the specificity of qPCR primers and qPCR product size, qPCR products were recovered from the Light Cycler® plates and visualized under ultraviolet light after 1.5% agarose gel electrophoresis. To further assess the nucleotide sequence variations, all qPCR products were sequenced with a BigDye Terminator v.3.1 Cycle Sequencing Kit (Applied Biosystems) on an ABI 3130 Genetic Analyzer (Applied Biosystems). The nucleotide sequences generated were aligned with each other using ClustalX2.0 (http://clustal.org/) for direct comparison of data among specimens.

### Nucleotide sequence accession

Representative nucleotide sequences generated in this study were deposited in GenBank under accession numbers MH647709-MH647717.

## Results

### Amplification efficiency of mitochondrial qPCR

**Fig. 1:**
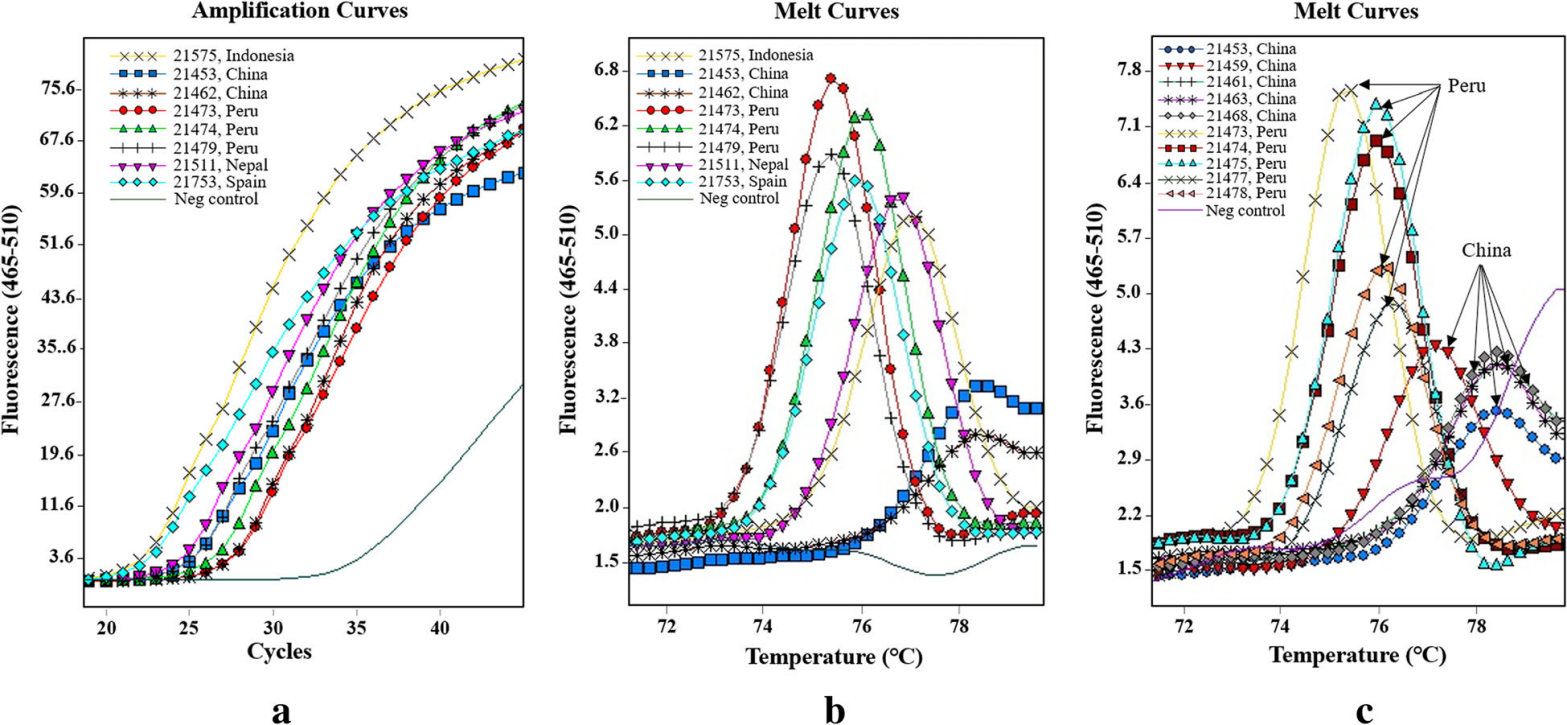
Amplification curves and melting curves of mitochondrial qPCR for *Cyclospora cayetanensis*. **a** Mitochondrial qPCR amplification curves of eight specimens from five countries. **b** Mitochondrial qPCR melt curves of eight specimens from five countries. **c** Mitochondrial qPCR melt curves of the specimens from China and Peru

In the qPCR analysis, all 36 specimens were identified as positive for *C. cayetanensis*, with Ct values ranging from 21.83 to 31.60 (Table 1). The negative control with reagent water, in contrast, produced Ct values above 35.0. The amplification curves generated were similar among specimens, except for differences in the Ct values (Fig. 1a, Table 1).

### Genotyping *C. cayetanensis* by melt curve analysis of qPCR products

In the mitochondrial qPCR, *C. cayetanensis* specimens were genotyped by melt curve analysis of the products at the end of the qPCR run. They produced various melt curve patterns with different Tm values, which indicated they belonged to different genotypes (Fig. 1b, Table 1). The Tm values of the Chinese specimens were obviously different from those of Peruvian specimens in the melt curve analysis (Fig. 1c, Table 1).

### Genotyping *C. cayetanensis* by gel electrophoresis analysis of qPCR products

**Fig. 2:**
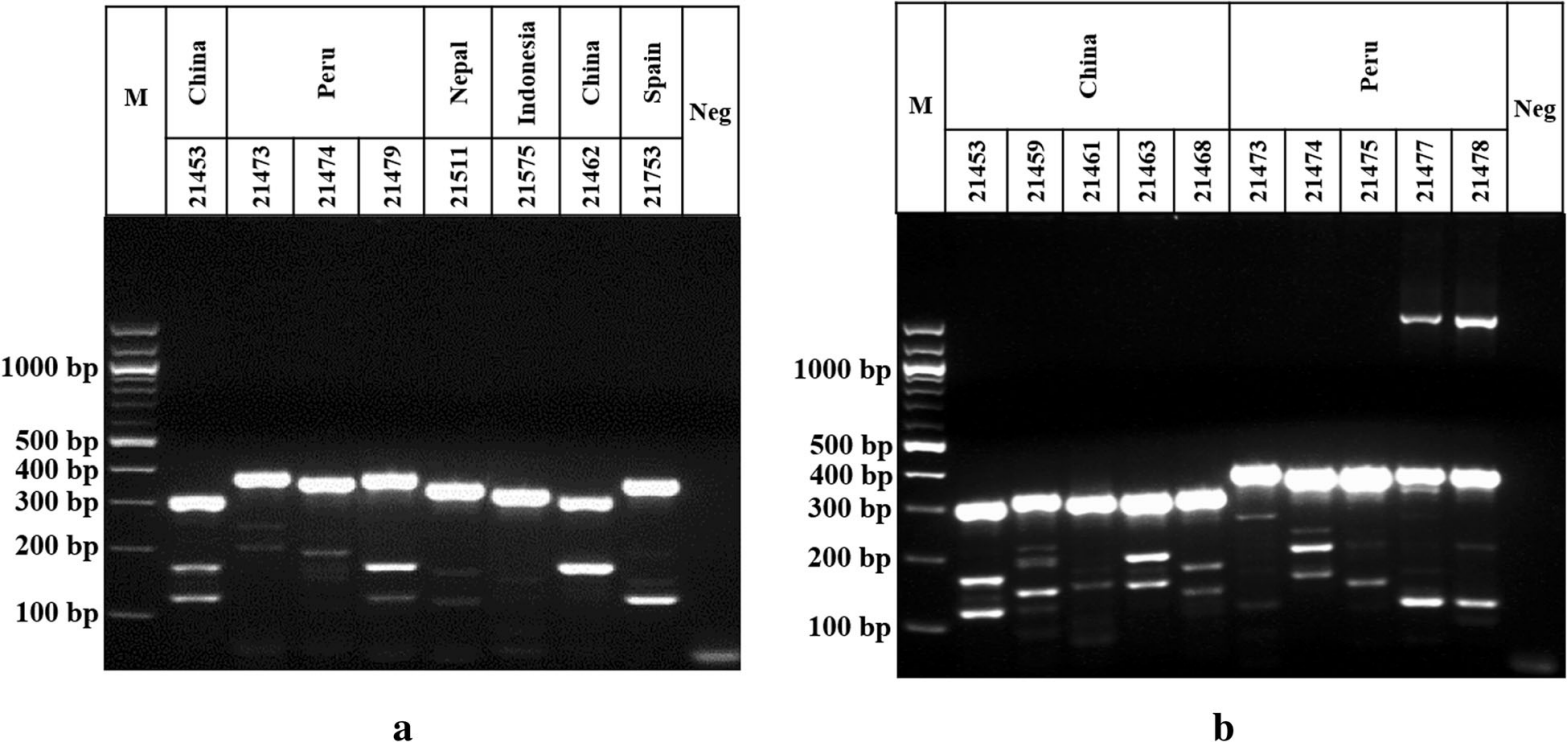
Length polymorphism of mitochondrial qPCR products from *Cyclospora cayetanensis*. **a** Electrophoretic profile of the mitochondrial qPCR products, Lane 1: 100-bp DNA ladder; Lanes 2–9: eight specimens from five countries; Lane 10: negative control. **b** Electrophoretic profile of the mitochondrial qPCR products from specimens from China and Peru, Lane 1: 100-bp DNA ladder; Lanes 2–6: specimens from China; Lanes 7–11: specimens from Peru; Lane 12: negative control

All qPCR products were analyzed by agarose gel electrophoresis. In this analysis, all specimens yielded the expected qPCR products along with some non-specific bands in PCR products from some specimens, which were all light in intensity and smaller in size (Fig. 2a). There was obvious length polymorphism among the amplicons in gel electrophoresis, with the presence of five types of qPCR products that differed slightly in length among eight specimens from five countries (Fig. 2a). This became especially obvious between the qPCR products of Chinese and Peruvian specimens (Fig. 2b).

### Genotyping *C. cayetanensis* by sequence analysis of qPCR products

**Fig. 3:**
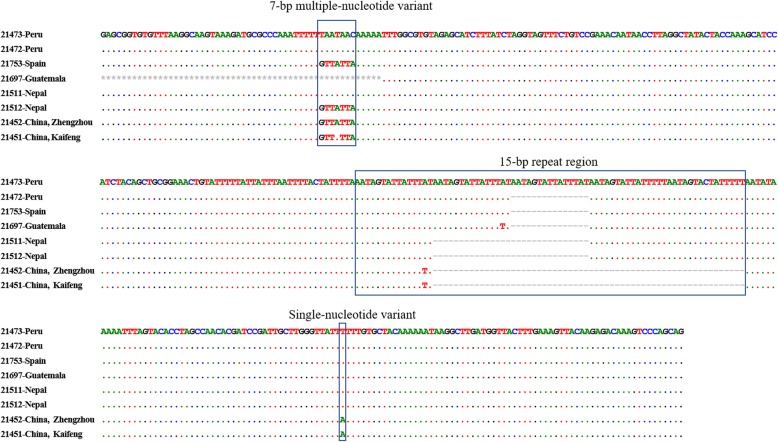
Sequence polymorphism among the nine genotypes generated from *Cyclospora cayetanensis* specimens by mitochondrial qPCR. Dots denote nucleotides identical to those in the first sequence of the sequence alignment, dashes denote nucleotide deletions and asterisks denote the unreadable nucleotides at the very beginning of the sequences

Of the 36 *C. cayetanensis*-positive specimens analyzed by the qPCR assay, 35 were sequenced successfully. The one specimen that failed to be sequenced contained mixed genotypes, indicated by the presence of underlying signals after the repeat region in sequencing trace files. There were nine genotypes detected from the 35 specimens (Table 1). The sequence polymorphism among these genotypes included one SNV, one 7-bp MNV, variations in the number of a 15-bp repeat (AAT AGT ATT ATT TAT, AAT AGT ATT ATT TTT or AAT AGT ACT ATT TTT), and length of the AT-rich link region (Fig. 3).

There were apparent geographical differences in DNA sequences of *C. cayetanensis* specimens (Table 1). All sequences generated from Chinese specimens had only one copy of the 15-bp repeat and the same type of the 7-bp MNV (GTTATTA), except for one specimen from Kaifeng, which had a variant of the 7-bp MNV (GTTTTTA) (Table 1, Fig. 3). In contrast, the two specimens from Indonesia had two copies of the 15-bp repeat; the three specimens from Nepal had three copies of the 15-bp repeat, but with different 7-bp MNVs (TAATAAC and GTTATTA); the Spanish specimen and the Guatemalan specimen had four copies of the 15-bp repeat; while the Peruvian specimens had four or five copies of the 15-bp repeat (Table 1, Fig. 3).

## Discussion

Although large cyclosporiasis outbreaks have occurred almost annually in North America and some European countries during the past 20 years, the prevention and control of cyclosporiasis outbreaks are still a major public health challenge [7]. The effectiveness of strategies to control the size and scope of an outbreak is largely determined by the speed and accuracy of outbreak and infection source identification. Therefore, it is important to timely genotype *C. cayetanensis* isolates from cluster cases and generate data that are helpful in the identification of the food vehicle involved and likely imported sources. In this study, we developed a qPCR targeting the polymorphic link region between copies of the mitochondrial genome. With melt curve or gel electrophoresis analysis of the qPCR products, genetic heterogeneity of *C. cayetanensis* among specimens can be quickly assessed. This would generate timely data on the diversity of parasites in specimens from cluster cases.

In theory, the qPCR developed in this study could be used for rapid assessment of genetic heterogeneity in *C. cayetanensis* among specimens in events of augmented number of cyclosporiasis cases. Previously, a MLST assay targeting five loci with short tandem repeats (STRs) was developed for *C. cayetanensis* [11]. The existence of extensive sequence polymorphism in addition to the expected variations in copy numbers of STRs allows high-resolution genotyping of *C. cayetanensis*. Nevertheless, the MLST assay relies heavily on DNA sequence analysis, which is time consuming and expensive. In contrast, the mitochondrial sequence-based qPCR can rapidly assess whether specimens analyzed belong to same or different genotypes based on melt curve patterns or by length polymorphism of amplicons. With the availability of the mitochondrial sequence-based qPCR, we can perform preliminary genotyping using sequence analysis of the qPCR products, while advanced genotyping can be offered by using the MLST assay. This would allow providing emergency responses in events of possible cyclosporiasis outbreaks.

The mitochondrial qPCR developed in this study appears to have high amplification efficiency. The high amplification efficiency of the mitochondrial sequence-based qPCR could be due to the high copy numbers (~500 copies) of the mitochondrial genome in *C. cayetanensis*. In contrast, the MLST genotyping assay generated sequence data at all five genetic loci from 34/64 specimens, leading to the successful genotyping of only 53.1% of the specimens under analysis. This was largely due to the presence of mixed genotypes from the USA specimens at one of the loci, leading to the failure in identifying *C. cayetanensis* genotypes for these specimens. This would not be an issue for melt curve- or gel electrophoresis-based genotyping. In the gel electrophoresis of the qPCR products, the expected qPCR bands were clear and differentiable. The non-specific bands, in contrast, were light in intensity and smaller in size. As a result, they did not affect the interpretation of typing results and DNA sequencing. Some of the non-specific amplification was probably due to excessive cycling (50 cycles).

The genotyping result of the mitochondrial qPCR has revealed significant geographical segregation of genotypes among the specimens under analysis. By Tm values in melt curve analysis and qPCR product sizes in gel electrophoresis analysis, most of the specimens from different countries can be differentiated. The two specimens from Spain and Guatemala, however, had similar melt curve patterns, probably because the Spanish patient could have acquired *C. cayetanensis* infection in South America. Similarly, one of the specimens from Nepal, 21511, also had a melt curve similar to the one generated from two Indonesian specimens.

The geographical differences in melt curve patterns among *C. cayetanensis* specimens was confirmed by sequence analysis of the qPCR products. In the latter, specimens from different countries mostly varied in the number of a 15-bp repeat sequence. In addition, specimens from China and Nepal differed from others by the nature of a 7-bp MNV. Significant sequence differences are also present between the Nepalese and Indonesian specimens with similar melt curve patterns (Fig 3).

There is a very good agreement in genotyping results between the mitochondrial qPCR and the nuclear sequence-based MLST assay. In a previous study using the MLST genotyping assay, most specimens from China clustered into one of two major groups, which are distinct from the groups formed by Peruvian specimens [11]. The advantage of the mitochondrial sequence-based qPCR is the significantly higher amplification efficiency and reduced impact of concurrence of mixed genotypes, resulting in significantly more specimens being successfully genotyped with this tool than the nuclear sequence-based MLST assay.

## Conclusions

In conclusion, a qPCR targeting the polymorphic link region of the mitochondrial genome of *C. cayetanensis* was developed. With melt curve or gel electrophoresis analysis, this method is able to rapidly assess the genetic heterogeneity among *C. cayetanensis* isolates. Genotyping and geographical source-tracking of the isolates could be further provided through DNA sequencing of the qPCR products. Nevertheless, there is a need of more studies using specimens from other geographical regions to validate the typing resolution and source-tracking ability of this mitochondrial genomic marker.

## Abbreviations

MLST: Multiple locus sequence typing
MNV: Multiple-nucleotide variant
qPCR: Quantitative polymerase chain reaction
SNVs: Single-nucleotide variants
